# Retrograde Flushing Followed by Slicing Float-Up as an Approach to Optimize Epididymal Sperm Recovery for the Purpose of Cryopreservation in Equids

**DOI:** 10.3390/ani12141802

**Published:** 2022-07-14

**Authors:** Giorgia Podico, Igor F. Canisso

**Affiliations:** Department of Veterinary Clinical Medicine, College of Veterinary Medicine, University of Illinois Urbana-Champaign, Urbana, IL 61801, USA; gpodico@illinois.edu

**Keywords:** cryopreservation, conservation

## Abstract

**Simple Summary:**

Harvesting epididymal sperm often represents the last opportunity to cryopreserve the breeding potential of a sire. Epididymal sperm harvesting is performed via retrograde flushing (RF) or slicing float-up (SF) of sperm into an extender. However, these techniques are not applied in combination unless RF results in poor yield. This study aimed to assess the yield and cryopreservation of epididymal sperm harvested via RF followed by SF in equids. The total sperm harvested by RF in donkeys resulted in the greatest yield, and horse epididymal harvesting via SF resulted in the fewest yield. The use of RF followed by SF resulted in 57% and 31% more sperm per harvest in the donkey and horse. Technique did not affect pre- and post-freezing sperm parameters. These results suggest that this could become a new standard approach in clinical practice.

**Abstract:**

This study aimed to assess the parameters of epididymal sperm harvested by retrograde flushing (RF) followed by slicing float-up (SF). Epididymides from donkeys (*n* = 18) and horses (*n* = 28) were subjected to RF with a freezing extender and then SF technique. The retrieved sperm after RF and SF was evaluated for volume, concentration, and total sperm and then cryopreserved separately. Post-thaw total motility (TM) and progressive motility (PM) were evaluated with CASA. Sperm membrane integrity (SMI) and mitochondrial membrane potential (MMP) were assessed with flow cytometry. Sperm concentration was greater in donkeys than horses (684 ± 62.9 vs. 494 ± 50.9 million sperm/mL) (*p* = 0.02). The total sperm harvested was lower in SF (3.6 ± 0.7 billion) than RF (10.4 ± 1.5 billion) and in horses (4.6 ± 0.8 billion) than in donkeys (10.7 ± 1.8 billion) (*p* < 0.05). RF followed by SF resulted in 57% and 31% more sperm per harvest in donkeys and horses. Results of TM and PM before freezing were not affected by technique or species (*p* > 0.05). Post-thawing SMI and MMP did not vary with technique or species (*p* > 0.05); TM and PM were not influenced by the technique or the species (*p* > 0.05) but by their interaction (*p* = 0.005). In conclusion, using RF followed by SF enhances sperm recovery without affecting cryopreservation in equids.

## 1. Introduction

Harvesting and cryopreserving epididymal sperm often represent the last opportunity to preserve the male breeding potential in mammals. In 1929, Walton reported the first successful epididymal sperm harvesting and insemination in rabbits [1]. Three decades later, Canadian investigators reported the birth of the first two horse foals resulting from epididymal sperm harvestings and cryopreservation [2]. However, minimal work was conducted on epididymal sperm cryopreservation in equids for nearly four decades. In the early 2000s, a wider acceptance of artificial insemination by many horse breeds and advances in semen processing led investigators to turn their focus on epididymal sperm cryobiology. In recent years, epididymal sperm harvesting and cryopreservation evolved remarkably and are widely used in all domesticated mammals [3,4,5,6,7,8] and have also been recently applied to captive and non-domesticated species such as giraffes [9], rhinoceros [10], and giant pandas [11].

Epididymal sperm harvesting is performed after castration, death, or euthanasia. The appropriate storage time and temperature vary with species; for instance, horses seem to tolerate better cold storage of the epididymides before harvesting than donkeys [8,12,13,14]. Anecdotally, retrograde flushing of the epididymal tail appears to be the technique most used in clinical practice across various domesticated species, and this certainly seems to be the case in equids [12,15,16]. The technique involves the removal of a small segment of the vas deferens and the tail of the epididymis away from the testis, then dissection of the convoluted epididymal duct and vas deferens to straighten up before canulation of the vas deferens and then retrograde flushing the tail of the epididymal with extender and air [7,15]. The extender used can be a semen cooling extender or a freezing extender [7]. If the first is used, centrifugation must be carried out after harvesting, while the latter requires no centrifugation and allows cryopreservation after adjustment of sperm concentration; both approaches result in similar post-thaw sperm parameters [7].

Retrograde flushing is considered the gold standard technique due to the high efficiency in sperm recovery with low blood contamination [7,15]. Conversely, slicing float-up is perceived to take a long time to perform and result in high blood contamination [7,15]. While performing retrograde flushing, the vas deferens can be cannulated with an intravenous catheter (12–21 G), butterfly, or tomcat catheter based on clinicians’ preferences, size of the vas deferens, or supply availability. This cannulation dictates the need for a patent and intact portion of the vas deferens immediately adjacent to the tail of the epididymal. However, if this segment of the vas deferens is damaged during castration/harvesting/dissection of the epididymides, or the animal has a tumor or adhesion in the area, an alternative technique such as slicing float-up needs to be applied. The technique involves slicing the tail of the epididymal in pieces and covering the slices with an extender, allowing sperm to float up into it. The content is filtered to remove the pieces of epididymal, and sperm is processed standardly pending whether a cooling or a freezing extender was used during float-up. If a freezing extender was used, no further processing other than adjustment of sperm concentration is necessary before cryopreservation, whereas if a cooling extender was used, centrifugation needs to be carried out to concentrate sperm and remove the excess of the cooling extender. Some clinicians prefer slicing float-up rather than retrograde flushing, regardless of if the epididymal tail is intact and patent. Other clinicians, such as the authors of this study, use float-up following retrograde flushing if the animal presents a poor sperm yield, or retrograde flushing cannot be performed as aforementioned. Maximizing the yield and quality of epididymal sperm can profoundly impact valuable sires and the preservation of endangered breeds and species. Up to now, the combination of these techniques has not been critically studied in mammals.

Therefore, the objectives of this study were to assess the yield and cryopreservation of epididymal sperm harvested via retrograde flushing followed by slicing float-up in equids. We hypothesized that performing the first technique followed by the latter would increase the total amount of sperm harvested without affecting the quality.

## 2. Materials and Methods

The present study was conducted at the Veterinary Teaching Hospital of the University of Illinois Urbana-Champaign, IL, between February 2020 and April 2022. Samples used in this study were derived from routine castrations (*n* = 9 donkeys and *n* = 13 horses) or euthanasia (*n* = 1 horse). Therefore, no oversight by Institutional Animal Care Unit Committee was necessary.

### 2.1. Animals and Study Design

**Figure 1:**
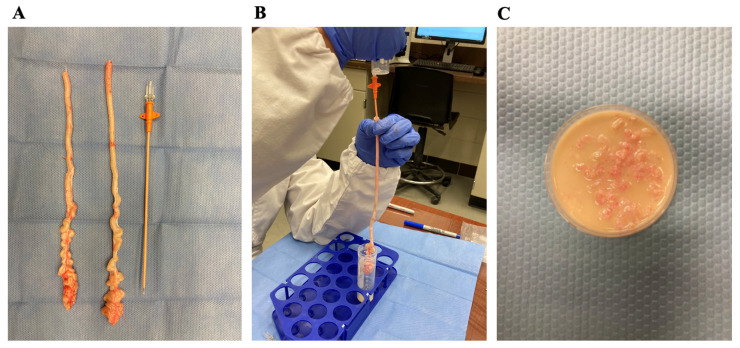
Epididymal sperm harvesting from a horse. The tail of the epididymis was dissected from the testis (**A**); the distal portion was straightened out, and an intravenous catheter was placed inside the lumen (**B**). Once the retrograde flushing of the tail was completed, the tail was minced into 1–2 mm pieces and submerged in an egg-yolk-based extender for 15–20 min (**C**).

Testes with attached epididymides and vas deferens were harvested from 14 stallions (6.3 ± 1.2 years old; ranging from 2 to 21 years old) and 9 jacks (5.2 ± 1.2 years old; ranging from 2 to 9 years old). The epididymal tails were dissected and weighed on a digital scale (FX 3000i, A&D Company Limited, Tokyo, Japan) (Figure 1A). Each tail of epididymis was kept at room temperature in a Petri dish moistened with Ringer’s Lactate solution until processing. The distal portion of each tail of the epididymis was carefully dissected to straighten a large portion of it; then, an intravenous catheter (16 G or 18 G) was used to cannulate the vas deferens toward the epididymal tail (Figure 1B) [7].

Retrograde epididymal flushing was performed with 5 to 10 milliliters of a commercially available egg-yolk-based freezing extender (Botucrio, Botupharma USA, Phoenix, AZ, USA) that were forced through the catheter into the vas deferens and epididymal tail. A small incision was made in the distal portion of the tail to ease the recovery of the sperm and extender in a conical tube [7]. After flushing with the extender, the syringe was loaded with 20–25 mL of air and pushed through the epididymal tail to maximize the recovery. The resulting volume, sperm concentration, and total sperm were recorded and used for further comparisons. Concentration was adjusted after this initial evaluation, and sperm motility parameters were assessed as described below.

After the retrograde flushing, each epididymal tail was submitted to the slicing and float-up technique. Each tail was minced with scissors into 1–2 mm pieces and kept on a Petri dish. Five to ten milliliters of the same extender were poured on top of the epididymal slices and left floating for 15–20 min at room temperature (Figure 1C). The fluid was recovered with a transfer pipette and filtered through gauze to remove gross debris. For further comparisons, the resulting sample was assessed for volume, sperm concentration, and total sperm. After this initial evaluation, concentration was adjusted, and sperm motility parameters were evaluated as described below.

### 2.2. Assessment of Sperm Concentration and Motility Parameters

The sperm concentration was determined with a Nucleocounter SP100 (ChemoMetec, Allerød, Denmark) following the manufacturer’s instructions. After retrograde flushing and slicing float-up, the total yield was determined by multiplying volume with sperm concentration. The results were used for comparison across techniques and species. Sperm motility parameters were assessed before and after freezing using a Computer-Assisted Sperm Analyzer (Spermvision, Minitube of America, Verona, WI, USA). Before sperm motility analysis, sperm concentration was adjusted to 50 million sperm/mL with the same freezing extender used for harvesting. After extension, the sample was mixed and incubated at 38 °C for 10 min before motility evaluation. An aliquot (8 µL) was placed on a heated slide and covered with a coverslip. At least 1000 sperm were assessed in 5–10 fields.

The manufacturer’s settings for equine sperm were used during the analyses and were as follows: static cell area 14–100 µm^2^, straightness threshold for progressive motility 90%, average path velocity threshold for static cell < 9.5 μm/s, light-emitting diode illumination intensity 180–255. Sperm kinematic parameters included total motility (TM, %), progressive sperm motility (PM, %), curvilinear velocity (VCL, µm/s), average path velocity (VAP, µm/s), and straight-line velocity (VSL, µm/s).

### 2.3. Sperm Freezing and Post-Thawing

After retrograde flushing and slicing float-up sperm were extended to 200 million sperm/mL, and samples obtained from either technique were loaded in 0.5 mL straws using an automated machine. Straws were placed in a cold room at 5 °C to equilibrate for 20 min. Thereafter, straws were placed 5 cm above liquid nitrogen vapor for 15 min and then plunged into liquid nitrogen. Straws were kept in liquid nitrogen for at least two weeks before analyses. Two straws cryopreserved with each technique were thawed at 38 °C for 60 s, and each sample was subjected to sperm motility analyses, plasma membrane integrity, and potential mitochondrial evaluations as described below.

### 2.4. Plasma Membrane Integrity and Mitochondrial Potential

The plasma membrane integrity and mitochondrial membrane potential were assessed using a spectral flow cytometer based on a published protocol [17]. The staining solutions were freshly prepared and frozen at −20 °C until used. The staining solution of Zombie Green dye (#423112 Biolegend, San Diego, CA, USA) was prepared with 100 μL of DMSO added to each vial of dye; similarly, MitoTracker Deep Red FM (M22426, Molecular Probes, Eugene, OR, USA) stock solution was prepared by adding DMSO to create a 10 μM working solution.

Sperm were resuspended to 3–5 million sperm/mL in PBS after centrifugation (600× *g* 10 min). Subsequently, a 100 μL aliquot of this solution was stained with both dyes (1 μL of Zombie Green and 1 μL MitoTracker Deep Red). After mixing, the sample was kept in the dark for 30 min at room temperature. Next, the sperm was washed (400× *g* 5 min), and each pellet was fixed with 250 μL of 2% buffered formalin and stored in the dark until flow cytometric evaluation. Before the acquisition with the flow cytometer, each sample was washed with 1 mL of PBS, centrifuged at 400× *g* for 5 min, and resuspended in PBS (250 μL). The analyses of the stained samples were conducted using a full-spectrum detector-based (filter-less) Cytek Aurora Flow Cytometer (Cytek Biosciences Inc., Fremont, CA, USA). At least 10,000 fluorescent gated events were recorded from each sample. Zombie Green was excited and detected with a 488 nm fluorescence detector, whereas MitoTracker Deep Red was excited with a 644/665 nm detector. Unstained and single-stained controls were used to unmix the signals. As previously described [17], four subpopulations of sperm were identified. The populations of sperm with intact (low Zombie Green signal) or damaged (high Zombie Green signal) plasma membranes were subdivided into low or high mitochondrial membrane potential based on the intensity of the signal given by Mitotracker Deep Red staining. Debris was manually excluded based on the minimal emitted fluorescence. Data from the flow cytometer were exported and analyzed with FlowJo (FlowJo v. 10 Software, Ashland, OR, USA). The percentage of sperm with the intact plasma membrane and the percentage of sperm with the intact plasma membrane and high mitochondrial potential were accounted for in comparisons across groups.

### 2.5. Statistical Analyses

Data analyses were carried out in R. Weight, concentration, and percentage of increase of sperm recovered were analyzed with a *t*-test. Sperm motility parameters, plasma membrane integrity, and mitochondrial potential were compared using a linear mixed model where the technique and the species were accounted for as fixed effects, and the animal was accounted for as random effects. Significance was set at *p* < 0.05. Data were presented as mean ± SEM.

## 3. Results

Donkey epididymis weighed 14.2 ± 2.3 g (ranging from 3.0–25.1 g) and did not differ from the horse epididymis, which weighed 18.5 ± 2.1 g and ranged from 10.4 to 28.0 g (*p* = 0.19). The total sperm harvested was affected by both the technique (*p* < 0.001) and the species (*p* = 0.007) but not by their interactions (*p* > 0.05). Donkey had greater sperm concentrations than horses (*p* = 0.02) (Table 1). The total sperm harvested by retrograde flushing in donkeys resulted in the greatest yield, and horse epididymal harvesting via slicing float-up resulted in the fewest yield. The use of retrograde flushing followed by slicing float-up resulted in 57% and 31% more sperm per harvest in the donkey and horse.

**Figure 2:**
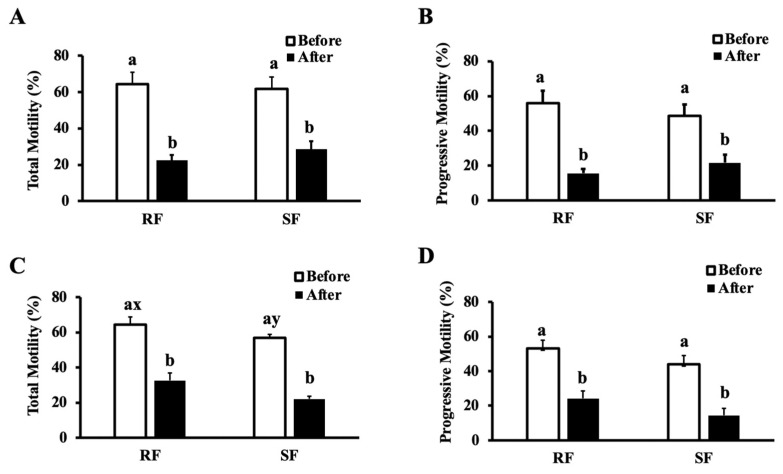
Total and progressive motility of epididymal sperm harvested by retrograde flushing of the tail followed by slicing float-up in donkeys (**A**,**B**) and horses (**C**,**D**). Motility parameters were assessed before and after freezing. Different superscript denote differences (*p* < 0.05).

Sperm harvested by retrograde flushing and slicing float-up had similar total and progressive motility for both horses and donkeys (*p* > 0.05) (Figure 2). Immediately after harvesting and before freezing, VAP was greater in retrograde flushing than the slicing float-up (*p* = 0.02) (Table 2), but it was similar between donkeys and horses (*p* = 0.41), and there was a tendency to have an interaction between the slicing float-up technique and the horse (*p* = 0.07). The VSL results before freezing were greater in retrograde flushing (*p* = 0.02) but were not influenced by the species (*p* = 0.18); an interaction between the horse and the slicing float-up technique was found (*p* = 0.03). Before freezing, results of VCL did not vary with the harvesting technique (*p* = 0.1), with species (*p* = 0.4), and there was no interaction between species and techniques (*p* > 0.05).

**Figure 3:**
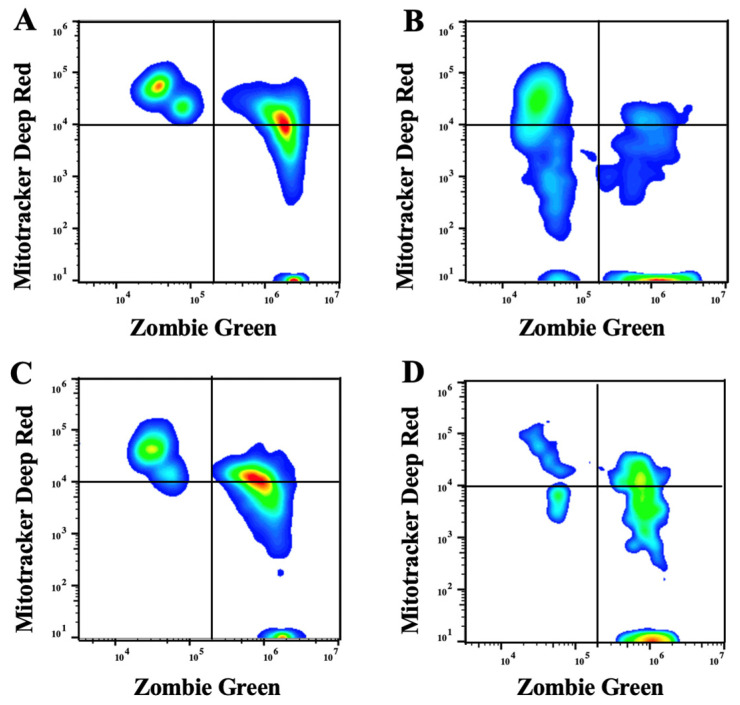
Representative plots of flow cytometric analyses from donkey (**A**,**B**) and horse (**C**,**D**) epididymal sperm. Sperm was obtained via retrograde flushing of the tail of the epididymis (**A**,**C**) followed by slicing float-up (**B**,**D**). Sperm was stained with Zombie Green and Mitotracker Deep Red to assess plasma membrane integrity and mitochondrial membrane potential. The left quadrants depict the subpopulation of intact sperm; the right quadrants represent the subpopulation of damaged sperm; the upper and lower quadrants identify the subpopulation of sperm with high and low mitochondrial membrane potential, respectively.

**Figure 4:**
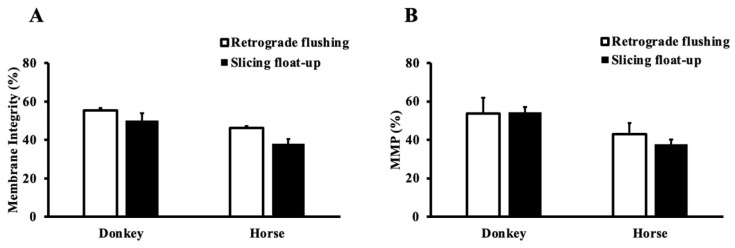
Sperm plasma membrane integrity (**A**) and mitochondrial membrane potential(MMP) (**B**) of frozen-thawed epidydimal sperm harvested via retrograde flushing of the tail of the epididymis followed by slicing float-up in the donkey and the horse. MMP represents the percentage of intact sperm with high mitochondrial membrane potential.

After thawing, the sperm membrane integrity and the percentage of intact sperm with high mitochondrial membrane potential were not affected by the technique or the species (*p* > 0.05) (Figure 3 and Figure 4). Post-thaw total and progressive motility and VAP did not vary with technique or the species (*p* > 0.05) but by their interaction (*p* = 0.005); the slicing float-up technique applied to the horse resulted in lower total motility (*p* < 0.05). The VSL was affected by the freezing (*p* < 0.001) and by the species (*p* < 0.001); VSL was lower in the stallion epididymal semen. Total and progressive motility, VAP, and VSL decreased after freezing (*p* < 0.001) (Table 2, Figure 2).

## 4. Discussion

This study was designed to demonstrate the usefulness of a method to maximize epididymal sperm yield and cryopreservation in equids. The approach described herein combines two epididymal sperm harvesting techniques (i.e., retrograde flushing and slicing float-up). Optimizing epididymal sperm harvesting can have a profound monetary and biological impact on breeding and conservation efforts. The method described herein aligns with this goal; it does not require special tools, and with minimal training, it can be readily applied to any clinical or farm setting to domesticated and non-domesticated species. Previously, this approach was used in our laboratory under specific circumstances, where retrograde flushing could not be applied, or it was applied and resulted in a perceived ineffective sperm recovery. This novel approach has the potential to become the standard method to harvest and cryopreserve epididymal sperm in equids and other domesticated mammals.

The superior sperm recovery obtained herein using retrograde flushing and then slicing float-up methods can have an even more impactful effect in the equid breeding industry than previously; nowadays, with the widespread use of intracytoplasmic sperm injection (ICSI) in horses [18] and more recently in donkeys [19], it has allowed maximum use of frozen sperm as 1000–10,000-fold less sperm is needed to result in a pregnancy in comparison with artificial insemination. As a result, many veterinary practices and clinicians are now simultaneously freezing sperm for use via artificial insemination and ICSI. The first is typically frozen at 100 million sperm/straw, whereas the latter is generally frozen at 2 to 10 million sperm/straw [18]. After freezing, one ICSI straw is typically sliced into multiple pieces; thus, a 2–10 million straw can be used in several rounds of ICSI. Thus, the enhanced sperm recovery of 31% and 57% in horses and donkeys could multiply by 100–1000 folds the reproductive potential of a sire. While the study did not evaluate fertility, nor was the sperm tried on ICSI, satisfactory results could likely have been obtained; however, this remains to be assessed.

After retrograde flushing, horse epididymal sperm via slicing float-up had slightly lower total and progressive motility than the first technique. This may suggest that the sperm cryopreserved with the latter could have lower fertility upon artificial insemination. On the other hand, it is possible that the sperm harvested with slicing float-up could be more suitable for ICSI, as the sperm used for this technique does not require satisfactory motility for good results [18].

The donkey sperm had lower post-thaw motility parameters than horses, the results are consistent with a recent study published by our laboratory [8]. The high plasma integrity and mitochondrial membrane potential in the presence of low motility parameters suggest that epididymal sperm motility is not activated by standard means such as using an egg yolk commercial extender such as Botucrio used herein or sperm centrifugation [7,8]. It remains to be determined how donkey epididymal sperm harvesting could be activated in vitro.

In equids, retrograde flushing of the epididymal tail is the preferred technique because of the perceived higher recovery and less blood contamination than the slicing float-up technique [7,15]. Herein, using retrograde flushing and then performing a slicing float-up technique enhanced the sperm yield remarkably in both species. The donkeys seemed to have benefited the most as the enlarged tail of the epididymis allows for larger storage of sperm [20], and likely for a similar reason, the epididymal sperm might be harder to recover with standard retrograde flushing, hence the superior recovery over horses in terms of percentages when combining both techniques. However, these findings could not be fully differentiated from the fact that donkeys produce more sperm than horses [21]. It has been demonstrated that the spermatogenic efficiency in donkeys is superior to other species of domestic mammals [21]. In addition, the spermatogenic cycle is shorter in the donkey (10.5 d) than in the horse (12.2 d); this, coupled with larger testes sizes, translates into a higher number of sperm being produced and recovered in donkeys than in horses [21].

The range of sperm concentration and total sperm was wide for both donkeys and horses, likely reflecting the wide range of ages and body sizes of the animals enrolled in the present study. Another factor that could not be accounted for in this study with a diverse population was the frequency of ejaculation, previous diseases, and seasonality. The animals of the present study were enrolled based on the occurrence of an elective castration or euthanasia, and only one of the stallions was known to be regularly collected when a sudden death occurred. There is scant literature on epididymal sperm harvesting in donkeys; however, a study from our laboratory had similar yields to that obtained herein by retrograde flushing the epididymal tail [8].

The present study highlighted that plasma membrane integrity is interconnected with mitochondrial membrane potential. Despite a non-significant difference, sperm plasma membrane integrity and mitochondrial were numerically higher in the donkey than in the horse. In alignment with this finding, studies showed that the midpiece region is more developed in the donkey than in the horse [21]. It is possible that if a larger number of animals were used, a statistical difference could have been detected.

Numerous European donkey breeds are at risk due to the reduced minimal number of animals in reproductive activity. The present study offers a method that can maximize the preservation of the genetics of a male that becomes ill, suddenly dies, or needs to be castrated for management reasons but could still be used as a genetic resource in the future. In the horse breeding industry, the genetic material of valuable sires can be maximized with the new technique described here and allow for excellent use of the genetic material of a valuable sire. More importantly, while the present study was conducted with equids, we have reasons to believe that the results obtained herein can easily translate into other species, such as carnivores and ruminants. Many breeds of dogs and cats, or even small ruminants, have a reduced vas deferens diameter, making cannulation of the vas deferens and retrograde flushing less effective than in equids; therefore, a combination of both techniques could benefit most of these species and other non-domestic species with similar anatomy. However, this remains to be tested in other species.

## 5. Conclusions

The use of retrograde flushing followed by slicing float-up of the tail of the epididymis increases the sperm yields without affecting the in vitro sperm quality in horses and donkeys. The approach combining both techniques described herein should become the new standard method to harvest and cryopreserve epididymal sperm in equids. It is likely that the techniques applied here can be used in other species with similar outcomes.

## Figures and Tables

**Table 1:** Concentration and total sperm harvested by retrograde flushing followed by slicing float-up of the tail of the epididymis in donkeys and horses.

	Donkey				Horse			
	Retrograde Flushing		Slicing Float-Up		Retrograde Flushing		Slicing Float-Up	
	Mean ± SEM	Range (Min–Max)	Mean ± SEM	Range (Min–Max)	Mean ± SEM	Range (Min–Max)	Mean ± SEM	Range (Min–Max)
Concentration (million/mL)	759 ± 84 ^ax^	56–1000	602 ± 92 ^ay^	2–942	660 ± 61 ^bx^	76–984	279 ± 42 ^by^	6–984
Total (billion)	14.8 ± 2.8 ^ax^	0.1–40	6.4 ± 1.3 ^ay^	7–14	7.5 ± 1.2 ^bx^	0.7–25	1.9 ± 0.4 ^by^	0.2–25

Different superscripts denote differences between species (^ab^) and techniques (^xy^) are denoted with different superscripts when *p* < 0.05.

**Table 2:** Sperm kinematic parameters of donkey and horse epidydimal sperm harvested by retrograde flushing and slicing float-up before and after freezing.

	Donkey				Horse			
	Retrograde Flushing		Slicing Float-Up		Retrograde Flushing		Slicing Float-Up	
	before	after	before	after	before	after	before	after
**VAP**	67.4 ± 3.5	51.9 ± 3.1	60.0 ± 4.3	51.6 ± 4.3	62.7 ± 2.7	51.6 ± 2.8	63.7 ± 2.1	53.4 ± 1.5
**VSL**	53.3 ± 3.0	42.2 ± 3.0	47.4 ± 2.7	41.7 ± 4.1	47.4 ± 2.2	40.2 ± 2.3	49.9 ± 1.8	43.2 ± 1.3
**VCL**	129.5 ± 6.3	106.2 ± 7.4	122.0 ± 6.4	99.6 ± 7.1	122.3 ± 5.0	97.9 ± 5.3	125.0 ± 3.5	103.0 ± 3.3

**Abbreviations: VAP**, average path velocity (μm/s); **VSL**, straight-line velocity (μm/s); **VCL**, curvilinear velocity (μm/s).

## Data Availability

Not applicable.
